# A Mobile Post Anesthesia Care Unit Order Reminder System Improves Timely Order Entry

**DOI:** 10.1007/s10916-024-02079-7

**Published:** 2024-06-10

**Authors:** Jacob C. Clifton, Holly B. Ende, Chandramouli Rathnam, Robert E. Freundlich, Warren S. Sandberg, Jonathan P. Wanderer

**Affiliations:** 1 Department of Anesthesiology, VAPIR Division, Vanderbilt University Medical Center, Nashville, TN USA; 2 Department of Biomedical Informatics, Vanderbilt University Medical Center, Nashville, TN USA

**Keywords:** PACU, CPOE, Reminders

## Abstract

Transition to the postanesthesia care unit (PACU) requires timely order placement by anesthesia providers. Computerized ordering enables automated order reminder systems, but their value is not fully understood. We performed a single-center, retrospective cohort study to estimate the association between automated PACU order reminders and primary outcomes (1) on-time order placement and (2) the degree of delay in placement. As a secondary post-hoc analysis, we studied the association between late order placement and PACU outcomes. We included patients with a qualifying postprocedure order from January 1, 2019, to May 31, 2023. We excluded cases transferred directly to the ICU, whose anesthesia provider was involved in the pilot testing of the reminder system, or those with missing covariate data. Order reminder system usage was defined by the primary attending anesthesiologist’s receipt of a push notification reminder on the day of surgery. We estimated the association between reminder system usage and timely order placement using a logistic regression. For patients with late orders, we performed a survival analysis of order placement. The significance level was 0.05. Patient (e.g., age, race), procedural (e.g., anesthesia duration), and provider-based (e.g., ordering privileges) variables were used as covariates within the analyses. Reminders were associated with 51% increased odds of order placement prior to PACU admission (Odds Ratio: 1.51; 95% Confidence Interval: 1.43, 1.58; p ≤ 0.001), reducing the incidence of late PACU orders from 17.5% to 12.6% (p ≤ 0.001). In patients with late orders, the reminders were associated with 10% quicker placement (Hazard Ratio: 1.10; 95% CI 1.05, 1.15; p < 0.001). On-time order placement was associated with decreased PACU duration (p < 0.001), decreased odds of peak PACU pain score (p < 0.001), and decreased odds of multiple administration of antiemetics (p = 0.02). An order reminder system was associated with an increase in order placement prior to PACU arrival and a reduction in delay in order placement after arrival.

## Introduction

The successful transition of care from the operating room to the post-anesthesia care unit (PACU) requires comprehensive communication between anesthesia, nursing, and surgical teams in the form of verbal hand offs as well as orders placed by the anesthesia team for necessary PACU therapies. Computerized provider order entry (CPOE) systems are frequently used for electronic placement of such orders and provide an opportunity for implementation of automated electronic reminders to encourage timely order placement. Since delays in PACU order placement can lead to delays in patient care and resulting adverse events or costs to the healthcare system, this type of reminder holds potential for improving patient outcomes.

Numerous studies have demonstrated the positive implications of CPOE in different clinical practice environments, showing that effective implementation can result in reduced errors, direct savings to the healthcare system, improved patient safety and outcomes, and decreased response and turnaround times [1–12]. However, limited data are available to study the implications of CPOE systems in the PACU setting, and specifically how reminder systems might provide value in supporting compliance with timely PACU order placement.

To mitigate delays in PACU order placement, a mobile electronic health record (EHR) reminder system was implemented at our institution in 2021. This study's aim was to estimate the association between the PACU mobile reminder system and the timeliness of PACU order placement and the degree of delay in care. We hypothesized that implementation of this reminder system would result in a greater proportion of patients having orders placed prior to arrival in PACU and more rapid order entry for those who did not have orders placed prior to arrival. Secondarily, we also studied the association of late order entry with various PACU outcomes.

## Methods

### Study Design/Sample

We performed a retrospective observational analysis, including subjects with an anesthetic care record from our institution’s main operating rooms who received a qualifying postprocedure order from January 1, 2019, to May 31, 2023.It is possible that anesthesia providers anticipated ICU transfers for sicker patients with higher case urgency, and thus the normal clinical flow for these cases was altered. For this reason, we excluded subjects admitted directly from the operating room (OR) to the intensive care unit (ICU). We also excluded those whose primary anesthesia attending provider participated in the pilot testing of the reminder system, and those with missing covariate data. This study was approved with a waiver for written, informed consent from the Human Subjects Research Protections Program at Vanderbilt University Medical Center and adheres to the guidelines provided in the STROBE (Strengthening the Reporting of Observational studies in Epidemiology) statement [13].

### Variables

Mobile reminder system usage was determined based on the EHR audit log of 'OR Closing’ push notifications sent to providers opted into the system and was defined for each case as “positive” (i.e., a reminder was sent for this case) if the anesthesia provider received a relevant reminder on the same day as the surgical case. The alerts were implemented using Epic System’s Haiku application (Verona, WI) and were designed to deliver a push notification to the attending anesthesiologist’s smartphone or tablet if PACU orders were not placed by the time that surgical closure began. Providers could opt out of push notifications by adjusting their application settings, or by choosing to not install the Haiku application on their personal smartphone or tablet. The study's main outcomes were (1) the incidence of patients arriving in the PACU without orders and (2) the degree of delay in order placement for those patients without orders present on arrival. To analyze the latter, we created a two-part survival variable to define (1) did patient have their order placed within 90 min of PACU start (Yes/No) and (2) minutes from PACU start to order placement. Patients who did not have their order placed within the first 90 min of their stay in PACU were considered negative for PACU order placement (i.e., lost to follow-up). This follow-up period was selected based on the distribution of time from PACU start to order placement for cases with late orders. Secondary outcomes were defined as (1) total PACU length of stay (LOS) in minutes, (2) ≥ 2 administrations of PACU antiemetics (promethazine, ondansetron, or haloperidol), and (3) peak PACU pain score.

### Data Collection Methods

Data on the relevant exposure, outcomes, and covariates were electronically extracted from Clarity, a relational database populated nightly from the EHR. Covariates included patient demographics (patient age, sex, self-reported race, height, and weight), procedural variables (American Society of Anesthesiologists’ [ASA] physical status classification, case level, and anesthesia duration), and three separate provider-based variables indicating if (1) the primary attending anesthesiologist ever changed, (2) the in-room provider ever changed, or (3) an in-room provider eligible to place orders was ever present during the case.

### Data Analyses

Demographic and procedural variables were summarized with median and interquartile range for continuous variables and with counts and percentages for categorical variables. Univariate groupwise comparisons were performed using the Chi-Squared test for categorical variables and Kruskal-Wallis Rank Sum test for continuous variables.

We first tested the univariate association between the PACU mobile reminder system and the incidence of patients arriving in the PACU with orders. We then estimated this association, adjusting for patient, procedural, and provider-based covariates, using a logistic regression. Next, we filtered out patients who did not have their order placed prior to PACU start and used a Cox Proportional Hazards survival model to analyze the association between the reminder system and degree of additional delay in order placement. Odds ratios (ORs) with 95% confidence intervals (CIs) quantified the effect of PACU mobile reminder system usage on the odds of arrival in PACU with orders already placed. Hazard ratios (HR) with 95% CI were reported for the association between the mobile reminder and the hazard, or the instantaneous rate of order placement. For numeric covariates, odds/hazard ratios were calculated by taking the difference in odds/hazard between the 25th and 75th percentile of the variable. Survival curves were plotted to visualize the difference in survival rates across patients who did and did not utilize the reminder system. ANOVA was utilized to test the significance of model terms. The significance level for model terms was set at 5%. Optimism-corrected concordance statistics were reported as metrics of discriminative performance of each respective model (i.e., ability of model to accurately distinguish between patients with and without the outcome of interest). Calibration plot assessed the agreement between predicted and observed outcomes, corrected for optimism (i.e., adjustment for overfitting). Restricted cubic splines were utilized to relax the linearity assumption of continuous covariates. All analyses were performed with R (Version 4.1.2; R Core Team; Vienna, Austria) and libraries used include tidyverse, knitr, Hmisc, rms, tableone, RODBC, DBI, odbc, pROC, survival, survminer, ggpubr, car, and plotly.

### Post-hoc Secondary Analysis

As a post-hoc secondary analysis, we assessed the association between order placement prior to PACU admission and secondary PACU outcomes (PACU duration, administration of antiemetics, and peak pain score). Due to its distribution, we performed a linear regression of a logarithmic transformation of the total PACU length of stay variable. Logistic regression was utilized to study the second administration of antiemetics, and a proportional odds logistic regression was used to study the ordered peak PACU pain score outcome.

## Results

**Fig. 1:**
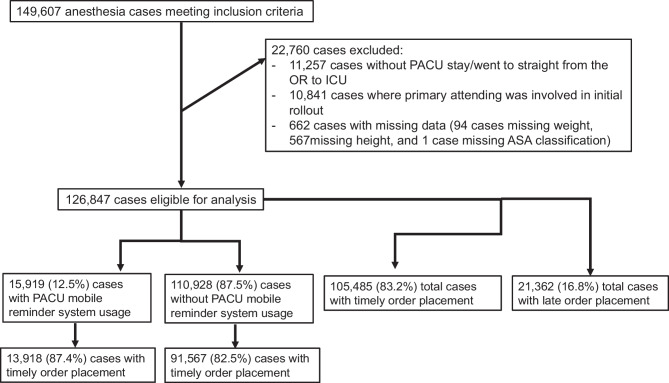
Inclusion-exclusion flow chart

Of the 149,607 records that met our inclusion criteria, 11,257 cases went straight from the OR to the ICU, 10,841 cases had a primary attending who was involved in the initial pilot testing of the reminder system, and 662 cases were excluded for missing data (Fig. 1). There were thus 126,847 cases that were eligible for analysis. In 12.5% (n = 15,919) of cases, the primary attending provider received a push notification reminder on the day of surgery (Table 1). Overall, 88 (44.9%) attending providers received at least one ‘OR Closing’ push notification. Use of the mobile reminder system was associated with a reduced incidence of missing PACU orders on admission (12.6% vs. 17.5%; p < 0.001) and orders that were placed sooner on average (p < 0.001) (Table 2). The rate of order placement prior to PACU start was 83.2% (n = 105,485). Compared to those whose orders were late, cases with timely order placement had on average higher rates of mobile reminder system usage by the attending provider (13.2% vs. 9.4%; p < 0.001) (Table 3).

**Table 1:** Sample descriptive statistics

	**CASES MEETING INCLUSION CRITERIA**^**b**^	**CASES MEETING EXCLUSION CRITERIA**^**b**^	**ANALYZED CASES**	**P-VALUE**^**c**^
TOTAL PATIENTS (%)	149,607	22,760 (15.2)	126,847 (84.8)	
ORDER PLACED BEFORE PACU START (%)	115,449 (77.2)	9,964 (43.8)	105,485 (83.2)	< 0.001
ORDER PLACED WITHIN 90 MINUTES OF PACU START (%)	148,951 (99.6)	22,641 (99.5)	126,310 (99.6)	0.04
MINUTES FROM PACU START TO ORDER PLACEMENT^a^ (MEDIAN [IQR])	-90 [-181, -23]	-97 [183, -32]]	-89 [-181, -22]	< 0.001
TOTAL PACU DURATION, MINUTES (MEDIAN [IQR])	122 [81, 204]	123 [83, 208]	122 [81, 203]	0.003
2ND+ LINE ANTIEMETICS (%)	7,375 (4.9)	601 (2.6)	6,774 (5.3)	0.612
PEAK PACU PAIN SCORE (%)				< 0.001
0 (NO PAIN)	37,656 (27.6)	2,943 (25.9)	34,713 (27.8)	
1	2,206 (1.6)	195 (1.7)	2,011 (1.6)	
2	5,256 (3.9)	466 (4.1)	4,790 (3.8)	
3	6,739 (4.9)	556 (4.9)	6,183 (5.0)	
4	6,885 (5.1)	560 (4.9)	6,325 (5.1)	
5 (MODERATE PAIN)	9,723 (7.1)	740 (6.5)	8,983 (7.2)	
6	9,106 (6.7)	776 (6.8)	8,330 (6.7)	
7	16,796 (12.3)	1,381 (12.2)	15,415 (12.3)	
8	17,290 (12.7)	1,516 (13.4)	15,774 (12.6)	
9	8,149 (6.0)	712 (6.3)	7,437 (6.0)	
10 (WORST POSSIBLE PAIN)	16,433 (12.1)	1,502 (13.2)	14,931 (12.0)	
ATTENDING PROVIDER MOBILE REMINDER SYSTEM USAGE (%)	20,224 (13.5)	4,305 (18.9)	15,919 (12.5)	< 0.001
ATTENDING PROVIDER CHANGE DURING CASE (%)	20,012 (13.4)	4,462 (19.6)	15,550 (12.3)	< 0.001
IN-ROOM PROVIDER CHANGE DURING CASE (%)	17,707 (11.8)	4,205 (18.5)	13,502 (10.6)	< 0.001
IN-ROOM ORDERING PROVIDER PRESENT DURING CASE (%)	120,971 (80.9)	18,057 (79.3)	102,914 (81.1)	< 0.001
IN-ROOM ORDERING PROVIDER CHANGE DURING CASE (%)	11,808 (7.9)	2,420 (10.6)	9,388 (7.4)	< 0.001
ANESTHESIA DURATION, MINUTES (MEDIAN [IQR])	142 [90, 214]	154 [99, 238]	140 [89, 210]	< 0.001
SEX = MALE (%)	77,208 (51.6)	12,487 (54.9)	64,721 (51.0)	< 0.001
AGE YEARS (MEDIAN [IQR])	57 [42, 68]	56 [41, 67]	57 [42, 68]	< 0.001
RACE (%)				< 0.001
WHITE	124,632 (83.3)	18,272 (80.3)	106,360 (83.8)	
AMERICAN INDIAN OR ALASKA NATIVE	289 (0.2)	36 (0.2)	253 (0.2)	
ASIAN	1,616 (1.1)	224 (1.0)	1,392 (1.1)	
BLACK OR AFRICAN AMERICAN	18,011 (12.0)	3,208 (14.1)	14,803 (11.7)	
UNKNOWN OR OTHER	5,059 (3.4)	1,020 (4.5)	4,039 (3.2)	
PATIENT HISPANIC (%)				
HISPANIC OR LATINO/A	5,243 (3.5)	937 (4.1)	4,306 (3.4)	< 0.001
NOT HISPANIC OR LATINO/A	3 (0.0)	1 (0.0)	2 (0.0)	
UNKNOWN	144,361 (96.5)	21,822 (95.9)	122,539 (96.6)	
WEIGHT KG (MEDIAN [IQR])	83.9 [70.3, 99.8]	82.6 [69.4, 99.8]	83.9 [70.3, 99.8]	< 0.001
HEIGHT CM (MEDIAN [IQR])	170.2 [162.6, 180.3]	172.7 [162.6, 180.3]	170.2 [162.6, 180.3]	< 0.001
BMI (MEDIAN [IQR])	28.2 [24.3, 33.2]	28.0 [24.0, 33.0]	28.3 [24.4, 33.3]	< 0.001
ASA STATUS (%)				< 0.001
1/2	53,440 (35.7)	6,637 (29.2)	46,803 (36.9)	
3	86,696 (57.9)	13,354 (58.7)	73,342 (57.8)	
4/5	9,470 (6.3)	2,768 (12.2)	6,702 (5.3)	
PATIENT CLASS				< 0.001
SAME DAY PROCEDURE	78,529 (52.5)	6,311 (27.7)	72,218 (56.9)	
INPATIENT	64,371 (43.0)	15,681 (68.9)	48,690 (38.4)	
OBSERVATION	6,207 (4.1)	704 (3.1)	5,503 (4.3)	
OTHER	500 (0.3)	64 (0.3)	436 (0.3)	
CASE LEVEL				< 0.001
ELECTIVE	144,571 (96.6)	21,108 (92.7)	123,463 (97.3)	
LEVEL 1 – MOST URGENT MEDICAL NEED	907 (0.6)	434 (1.9)	473 (0.4)	
LEVEL 2 – URGENT MEDICAL NEED	2,974 (2.0)	939 (4.1)	2,035 (1.6)	
LEVEL 3 – LEAST URGENT MEDICAL NEED	1,028 (0.7)	257 (1.1)	771 (0.6)	
LEVEL 4 – NEXT AVAILABLE REGULARLY SCHEDULED TIME	127 (0.1)	22 (0.1)	105 (0.1)	
SURGICAL SERVICE				< 0.001
ORTHOPEDIC	28,056 (18.8)	4,967 (21.8)	23,089 (18.2)	
OTOLARYNGOLOGY/H&N	19,439 (13.0)	2,389 (10.5)	17,050 (13.4)	
UROLOGY	16,836 (11.3)	1,517 (6.7)	15,319 (12.1)	
GENERAL SURGERY	13,665 (9.1)	1,305 (5.7)	12,360 (9.7)	
GENERAL ONCOLOGY SURGERY	10,414 (7.0)	935 (4.1)	9,479 (7.5)	
NEUROSURGERY/NEURO INTERVENTIONAL	11,584 (7.7)	2,753 (12.1)	8,831 (6.9)	
PLASTIC SURGERY	8,767 (5.9)	1,850 (8.1)	6,917 (5.5)	
OPHTHALMOLOGY	6,713 (4.5)	590 (2.6)	6,123 (4.8)	
PULMONARY	5,979 (4.0)	781 (3.4)	5,198 (4.1)	
VASCULAR	5,972 (4.0)	1,080 (4.7)	4,892 (3.9)	
OTHER	22,183 (14.8)	4,593 (20.2)	17,590 (13.9)	

^a^Negative values indicate that order placement occurred before PACU start

^b^Indicates that if missing data is present, then they are not included in the calculations

^c^p-values for groupwise comparisons are generated by the Chi-Squared test for categorical variables and Kruskal–Wallis Rank Sum test for continuous variables

**Table 2:** Sample descriptive statistics, stratified by exposure

	**ANALYZED CASES**	**NO MOBILE REMINDER SYSTEM**	**YES MOBILE REMINDER SYSTEM**	**P-VALUE**^**b**^
TOTAL PATIENTS (%)	126,847	110,928 (87.5)	15,919 (12.5)	
ORDER PLACED BEFORE PACU START (%)	105,485 (83.2)	91,567 (82.5)	13,918 (87.4)	< 0.001
ORDER PLACED WITHIN 90 MINUTES OF PACU START (%)	126,310 (99.6)	110,430 (99.6)	15,880 (99.8)	< 0.001
MINUTES FROM PACU START TO ORDER PLACEMENT^a^ (MEDIAN [IQR])	-89 [-181, -22]	-88 [-177, -21]	-103 [-210, -30]	< 0.001
TOTAL PACU DURATION, MINUTES (MEDIAN [IQR])	122 [81, 203]	121 [80, 202]	126 [84, 214]	< 0.001
2ND+ LINE ANTIEMETICS (%)	6,774 (5.3)	5,887 (5.3)	887 (5.6)	0.17
PEAK PACU PAIN SCORE (%)				0.016
0 (NO PAIN)	34,713 (27.8)	30,461 (27.9)	4,252 (26.9)	
1	2,011 (1.6)	1,732 (1.6)	279 (1.8)	
2	4,790 (3.8)	4,190 (3.8)	600 (3.8)	
3	6,183 (5.0)	5,417 (5.0)	766 (4.8)	
4	6,325 (5.1)	5,510 (5.1)	815 (5.2)	
5 (MODERATE PAIN)	8,983 (7.2)	7,892 (7.2)	1,091 (6.9)	
6	8,330 (6.7)	7,298 (6.7)	1,032 (6.5)	
7	15,415 (12.3)	13,457 (12.3)	1,958 (12.4)	
8	15,774 (12.6)	13,673 (12.5)	2,101 (13.3)	
9	7,437 (6.0)	6,478 (5.9)	959 (6.1)	
10 (WORST POSSIBLE PAIN)	14,931 (12.0)	12,961 (11.9)	1,970 (12.5)	
ATTENDING PROVIDER MOBILE REMINDER SYSTEM USAGE (%)	15,919 (12.5)	0 (0.0)	15,919 (100.0)	-
ATTENDING PROVIDER CHANGE DURING CASE (%)	15,550 (12.3)	13,759 (12.4)	1,791 (11.3)	< 0.001
IN-ROOM PROVIDER CHANGE DURING CASE (%)	13,502 (10.6)	11,953 (10.8)	1,549 (9.7)	< 0.001
IN-ROOM ORDERING PROVIDER PRESENT DURING CASE (%)	102,914 (81.1)	90,526 (81.6)	12,388 (77.8)	< 0.001
IN-ROOM ORDERING PROVIDER CHANGE DURING CASE (%)	9,388 (7.4)	8,340 (7.5)	1,048 (6.6)	< 0.001
ANESTHESIA DURATION, MINUTES (MEDIAN [IQR])	140 [89, 210]	139 [89, 210]	141 [90, 212]	0.003
SEX = MALE (%)	64,721 (51.0)	56,727 (51.1)	7,994 (50.2)	0.033
AGE YEARS (MEDIAN [IQR])	57 [42, 68]	57 [42, 68]	58 [42, 69]	< 0.001
RACE (%)				< 0.001
WHITE	106,360 (83.8)	93, 178 (84.0)	13,182 (82.8)	
AMERICAN INDIAN OR ALASKA NATIVE	253 (0.2)	211 (0.2)	42 (0.3)	
ASIAN	1,392 (1.1)	1,201 (1.1)	191 (1.2)	
BLACK OR AFRICAN AMERICAN	14,803 (11.7)	12,876 (11.6)	1,927 (12.1)	
UNKNOWN OR OTHER	4,039 (3.2)	3,462 (3.1)	577 (3.6)	
PATIENT HISPANIC (%)				
HISPANIC OR LATINO/A	4,306 (3.4)	3,612 (3.3)	694 (4.4)	< 0.001
NOT HISPANIC OR LATINO/A	2 (0.0)	1 (0.0)	1 (0.0)	
UNKNOWN	122,539 (96.6)	107,315 (96.7)	15,224 (95.6)	
WEIGHT KG (MEDIAN [IQR])	83.9 [70.3, 99.8]	83.9 [70.3, 99.8]	83.5 [70.0, 99.8]	0.421
HEIGHT CM (MEDIAN [IQR])	170.2 [162.6, 180.3]	170.2 [162.6, 180.3]	170.2 [162.6, 180.3]	0.113
BMI (MEDIAN [IQR])	28.3 [24.4, 33.3]	28.3 [24.4, 33.3]	28.2 [24.3, 33.3]	0.664
ASA STATUS (%)				< 0.001
1/2	46,803 (36.9)	41,316 (37.2)	5,487 (34.5)	
3	73,342 (57.8)	63,810 (57.5)	9,532 (59.9)	
4/5	6,702 (5.3)	5,802 (5.2)	900 (5.7)	
PATIENT CLASS				0.001
SAME DAY PROCEDURE	72,218 (56.9)	63,345 (57.1)	8,873 (55.7)	
INPATIENT	48,690 (38.4)	42,394 (38.2)	6,296 (39.6)	
OBSERVATION	5,503 (4.3)	4,792 (4.3)	711 (4.5)	
OTHER	436 (0.3)	397 (0.4)	39 (0.2)	
CASE LEVEL				0.001
ELECTIVE	123,463 (97.3)	107,925 (97.3)	15,538 (97.6)	
LEVEL 1 – MOST URGENT MEDICAL NEED	473 (0.4)	410 (0.4)	63 (0.4)	
LEVEL 2 – URGENT MEDICAL NEED	2,035 (1.6)	1,805 (1.6)	230 (1.4)	
LEVEL 3 – LEAST URGENT MEDICAL NEED	771 (0.6)	683 (0.6)	88 (0.6)	
LEVEL 4 – NEXT AVAILABLE REGULARLY SCHEDULED TIME	105 (0.1)	105 (0.1)	0 (0.0)	
SURGICAL SERVICE				< 0.001
ORTHOPEDIC	23,089 (18.2)	20,185 (18.2)	2,904 (18.2)	
OTOLARYNGOLOGY/H&N	17,050 (13.4)	15,098 (13.6)	1,952 (12.3)	
UROLOGY	15,319 (12.1)	13,486 (12.2)	1,833 (11.5)	
GENERAL SURGERY	12,360 (9.7)	10,694 (9.6)	1,666 (10.5)	
GENERAL ONCOLOGY SURGERY	9,479 (7.5)	8,299 (7.5)	1,180 (7.4)	
NEUROSURGERY/NEURO INTERVENTIONAL	8,831 (6.9)	7,906 (7.1)	925 (5.8)	
PLASTIC SURGERY	6,917 (5.5)	6,022 (5.4)	895 (5.6)	
OPHTHALMOLOGY	6,123 (4.8)	5,484 (4.9)	639 (4.0)	
PULMONARY	5,198 (4.1)	4,385 (4.0)	813 (5.1)	
VASCULAR	4,892 (3.9)	4,137 (3.7)	755 (4.7)	
OTHER	17,590 (13.9)	15,232 (13.7)	2,358 (14.8)	

^a^Negative values indicate that order placement occurred before PACU start

^b^p-values for groupwise comparisons are generated by the Chi-Squared test for categorical variables and Kruskal-Wallis Rank Sum test for continuous variables

**Table 3:** Sample descriptive statistics, stratified by primary outcome

	**ANALYZED CASES**	**ORDER PLACED AFTER PACU START**	**ORDER PLACED BEFORE PACU START**	**P-VALUE**^**b**^
TOTAL PATIENTS (%)	126,847	21,362 (16.8)	105,485 (83.2)	
ORDER PLACED BEFORE PACU START (%)	105,485 (83.0)	0 (0.0)	105,485 (100.0)	-
ORDER PLACED WITHIN 90 MINUTES OF PACU START (%)	126,310 (99.6)	20,825 (97.5)	105,485 (100.0)	-
MINUTES FROM PACU START TO ORDER PLACEMENT^a^ (MEDIAN [IQR])	-89 [-181, -22]	13 [6, 27]	-115 [-204, -56]	< 0.001
TOTAL PACU DURATION, MINUTES (MEDIAN [IQR])	122 [81, 203]	125 [84, 201]	121 [80, 204]	< 0.001
2ND+ LINE ANTIEMETICS (%)	6,774 (5.3)	1,155 (5.4)	5,619 (5.3)	0.647
PEAK PACU PAIN SCORE (%)				< 0.001
0 (NO PAIN)	34,713 (27.8)	5,041 (24.0)	29,672 (28.6)	
1	2,011 (1.6)	302 (1.4)	1,709 (1.6)	
2	4,790 (3.8)	699 (3.3)	4,091 (3.9)	
3	6,183 (5.0)	930 (4.4)	5,253 (5.1)	
4	6,325 (5.1)	1,011 (4.8)	5,314 (5.1)	
5 (MODERATE PAIN)	8,983 (7.2)	1,481 (7.0)	7,502 (7.2)	
6	8,330 (6.7)	1,423 (6.8)	6,907 (6.6)	
7	15,415 (12.3)	2,636 (12.5)	12,779 (12.3)	
8	15,774 (12.6)	3,010 (14.3)	12,764 (12.3)	
9	7,437 (6.0)	1,392 (6.6)	6,045 (5.8)	
10 (WORST POSSIBLE PAIN)	14,931 (12.0)	3,093 (14.7)	11,838 (11.4)	
ATTENDING PROVIDER MOBILE REMINDER SYSTEM USAGE (%)	15,919 (12.5)	2,001 (9.4)	13,918 (13.2)	< 0.001
ATTENDING PROVIDER CHANGE DURING CASE (%)	15,550 (12.3)	1,839 (8.6)	13,711 (13.0)	< 0.001
IN-ROOM PROVIDER CHANGE DURING CASE (%)	13,502 (10.6)	1,988 (9.3)	10,932 (10.4)	< 0.001
IN-ROOM ORDERING PROVIDER PRESENT DURING CASE (%)	102,914 (81.1)	16,320 (76.4)	86,594 (82.1)	< 0.001
IN-ROOM ORDERING PROVIDER CHANGE DURING CASE (%)	9,388 (7.4)	1,384 (6.5)	8,004 (7.6)	< 0.001
ANESTHESIA DURATION, MINUTES (MEDIAN [IQR])	140 [89, 210]	127 [82, 195]	142 [90, 213]	< 0.001
SEX = MALE (%)	64,721 (51.0)	11,126 (52.1)	53,595 (50.8)	0.002
AGE YEARS (MEDIAN [IQR])	57 [42, 68]	57 [42, 68]	57 [43, 68]	0.874
RACE (%)				0.112
WHITE	106,360 (83.8)	17,787 (83.3)	88,573 (84.0)	
AMERICAN INDIAN OR ALASKA NATIVE	253 (0.2)	39 (0.2)	214 (0.2)	
ASIAN	1,392 (1.1)	247 (1.2)	1,145 (1.1)	
BLACK OR AFRICAN AMERICAN	14,803 (11.7)	2,593 (12.1)	12,210 (11.6)	
UNKNOWN OR OTHER	4,039 (3.2)	696 (3.3)	3,343 (3.2)	
PATIENT HISPANIC (%)				
HISPANIC OR LATINO/A	4,306 (3.4)	700 (3.3)	3,606 (3.4)	0.265
NOT HISPANIC OR LATINO/A	2 (0.0)	1 (0.0)	1 (0.0)	
UNKNOWN	122,539 (96.6)	20,661 (96.7)	101,878 (96.6)	
WEIGHT KG (MEDIAN [IQR])	83.9 [70.3, 99.8]	83.6 [69.9, 99.8]	83.9 [70.3, 99.8]	0.021
HEIGHT CM (MEDIAN [IQR])	170.2 [162.6, 180.3]	170.2 [162.6, 180.3]	170.2 [162.6, 180.3]	0.522
BMI (MEDIAN [IQR])	28.3 [24.4, 33.3]	28.2 [24.2, 33.3]	28.3 [24.4, 33.3]	0.012
ASA STATUS (%)				< 0.001
1/2	46,803 (36.9)	7,222 (33.8)	39,581 (37.5)	
3	73,342 (57.8)	12,717 (59.5)	60,625 (57.5)	
4/5	6,702 (5.3)	1,423 (6.7)	5,279 (5.0)	
PATIENT CLASS				< 0.001
SAME DAY PROCEDURE	72,218 (56.9)	11,024 (51.6)	61,194 (58.0)	
INPATIENT	48,690 (38.4)	9,247 (43.3)	39,443 (37.4)	
OBSERVATION	5,503 (4.3)	985 (4.6)	4,518 (4.3)	
OTHER	436 (0.3)	106 (0.5)	330 (0.2)	
CASE LEVEL				< 0.001
ELECTIVE	123,463 (97.3)	20,540 (96.2)	102,923 (97.6)	
LEVEL 1 – MOST URGENT MEDICAL NEED	473 (0.4)	130 (0.6)	343 (0.3)	
LEVEL 2 – URGENT MEDICAL NEED	2,035 (1.6)	477 (2.2)	1,558 (1.5)	
LEVEL 3 – LEAST URGENT MEDICAL NEED	771 (0.6)	198 (0.9)	573 (0.5)	
LEVEL 4 – NEXT AVAILABLE REGULARLY SCHEDULED TIME	105 (0.1)	17 (0.1)	88 (0.1)	
SURGICAL SERVICE				< 0.001
ORTHOPEDIC	23,089 (18.2)	4,044 (18.9)	19,045 (18.1)	
OTOLARYNGOLOGY/H&N	17,050 (13.4)	2,502 (11.7)	14,548 (13.8)	
UROLOGY	15,319 (12.1)	2,867 (13.4)	12,452 (11.8)	
GENERAL SURGERY	12,360 (9.7)	1,830 (8.6)	10,530 (10.0)	
GENERAL ONCOLOGY SURGERY	9,479 (7.5)	1,058 (5.0)	8,421 (8.0)	
NEUROSURGERY/NEURO INTERVENTIONAL	8,831 (6.9)	1,920 (9.0)	6,911 (6.6)	
PLASTIC SURGERY	6,917 (5.5)	1,090 (5.1)	5,827 (5.5)	
OPHTHALMOLOGY	6,123 (4.8)	693 (3.2)	5,430 (5.1)	
PULMONARY	5,198 (4.1)	912 (4.3)	4,286 (4.1)	
VASCULAR	4,892 (3.9)	925 (4.3)	3,967 (3.8)	
OTHER	17,590 (13.9)	3,522 (16.5)	14,068 (13.3)	

^a^Negative values indicate that order placement occurred before after PACU start

^b^p-values for groupwise comparisons are generated by the Chi-Squared test for categorical variables and Kruskal-Wallis Rank Sum test for continuous variables

### Association Between PACU Mobile Reminder Usage and the Incidence of Timely PACU Order Placement

Order reminder system usage had a univariate association with increased rate of order placement prior to PACU admission (p < 0.001). Adjusting for patient, procedural, and provider-based variables, we found that PACU mobile reminder system usage was associated with 51% increased odds (OR: 1.51; 95% CI: 1.43, 1.58; p < 0.001) of timely PACU order placement (Table 4). Overall, there was no evidence of model overfit, but it displayed poor discriminative ability (optimism-corrected concordance statistic = 0.58).

**Table 4:** Primary adjusted analysis of association between incidence of arrival in PACU with order already placed and PACU mobile reminder system usage

**MODEL TERM**	**ODDS RATIO (95% CI)**	**P-VALUE**
PACU MOBILE REMINDER SYSTEM = YES	1.51 (1.43, 1.58)	≤ 0.001
ATTENDING CHANGE DURING CASE = YES	1.58 (1.49, 1.67)	≤ 0.001
IN-ROOM PROVIDER CHANGE DURING CASE = YES	0.85 (0.80, 0.89)	≤ 0.001
IN-ROOM ORDERING PROVIDER PRESENT EVER DURING CASE = YES	1.45 (1.40, 1.50)	≤ 0.001
ANESTHESIA DURATION, MINUTES (CHANGE IN ODDS FROM DURATION OF 89 TO 210)	1.16 (1.14, 1.19)	≤ 0.001
SEX = FEMALE (VS. MALE)	1.07 (1.02, 1.11)	0.01
AGE, YEARS (CHANGE IN ODDS FROM AGE OF 42 TO 68)	1.05 (1.02, 1.08)	≤ 0.001
RACE (REFERENCE = ‘WHITE’)		0.63
AMERICAN INDIAN OR ALASKA NATIVE	1.08 (0.76, 1.52)	
ASIAN	0.94 (0.81, 1.08)	
BLACK OR AFRICAN AMERICAN	0.97 (0.93, 1.02)	
UNKNOWN OR OTHER	0.97 (0.90, 1.06)	
HEIGHT, CM (CHANGE IN ODDS FROM HEIGHT OF 162.6 TO 180.3)	1.02 (0.99, 1.06)	0.48
WEIGHT, KG (CHANGE IN ODDS FROM WEIGHT OF 70.3 TO 99.8)	1.03 (1.01, 1.06)	0.02
ASA CLASS (REFERENCE = ‘1/2’)		≤ 0.001
3	0.82 (0.79, 0.85)	
4/5	0.65 (0.61, 0.70)	
CASE LEVEL (REFERENCE = ‘ELECTIVE’)		≤ 0.001
LEVEL 1 – MOST URGENT NEED	0.53 (0.43, 0.65)	
LEVEL 2 – URGENT MEDICAL NEED	0.68 (0.61, 0.75)	
LEVEL 3 – LEAST URGENT NEED	0.61 (0.52, 0.72)	
LEVEL 4 – NEXT AVAILABLE REGULARLY SCHEDULED TIME	1.10 (0.66, 1.86)	

### Association Between PACU Mobile Reminder Usage and Degree of Delay in Care

**Fig. 2:**
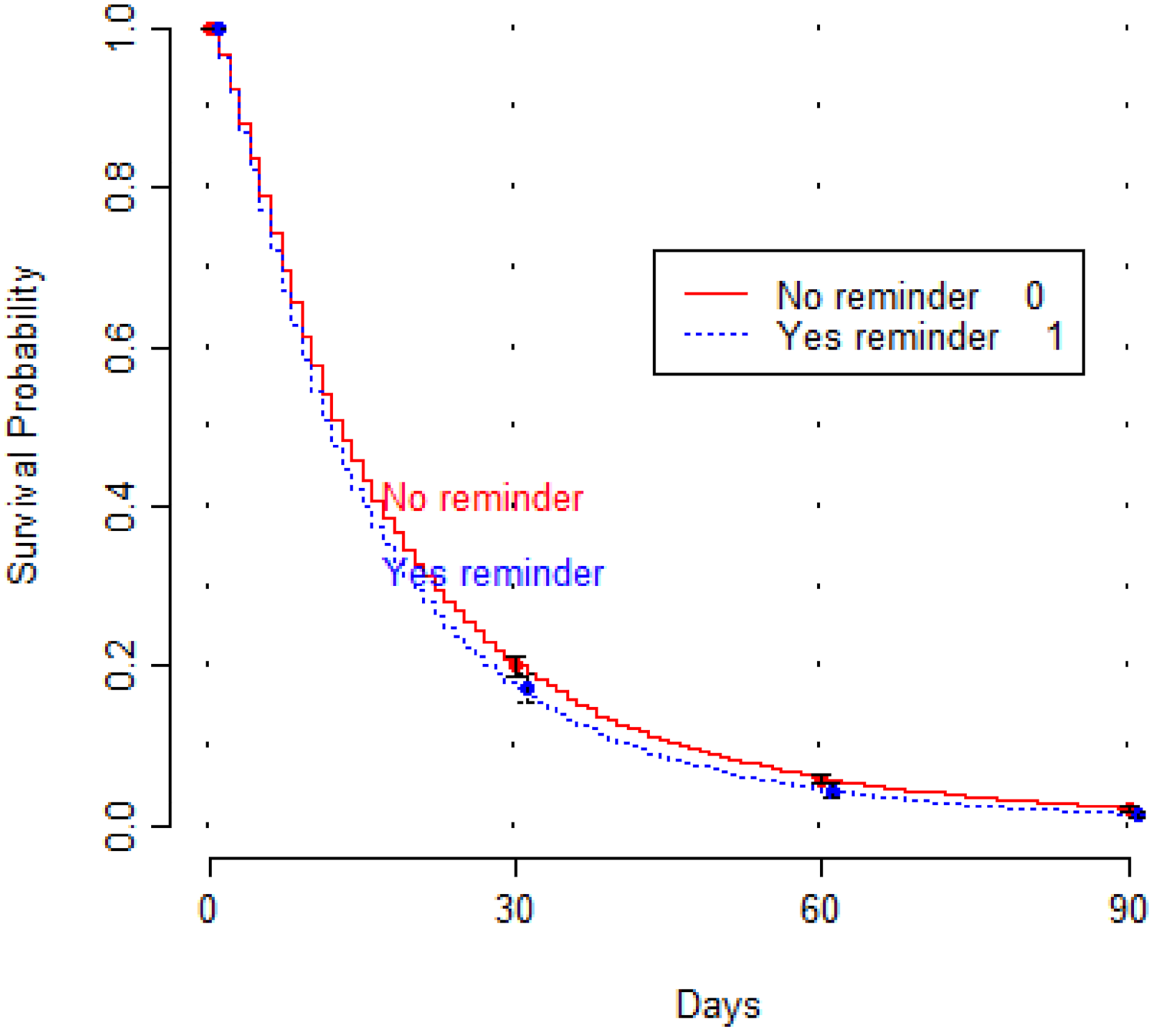
Survival curve

The records of 21,362 patients whose orders were not placed prior to PACU admission were analyzed to assess their degree of delay in order placement. There were 2,001 (9.4%) cases where the primary attending received a push notification on the day of surgery. Within this set of patients, usage of the PACU mobile reminder system was associated with 10% more rapid placement of a patient’s orders (HR: 1.10; 95% CI 1.05, 1.15; p < 0.001) (Table 5). Figure 2 shows that the delivery of a push notification reminder to the primary attending provider on the day of surgery was associated with reduced survival probability and, thus, were more likely to have order placement sooner than cases in which the primary attending did not receive a notification. The survival model displayed low corrective optimism adjustment for model performance (i.e., no evidence of overfit model) but displayed poor discriminative ability (optimism-corrected c-statistic = 0.54).

**Table 5:** Primary adjusted analysis of degree of delay in care in patients whose order was placed after PACU admission

**MODEL TERM**	**HAZARD RATIO (95% CI)**	**P-VALUE**
PACU MOBILE REMINDER SYSTEM = YES	1.10 (1.05, 1.15)	≤ 0.001
ATTENDING CHANGE DURING CASE = YES	0.91 (0.86, 0.96)	< 0.001
IN-ROOM PROVIDER CHANGE DURING CASE = YES	0.98 (0.93, 1.03)	0.45
IN-ROOM ORDERING PROVIDER PRESENT EVER DURING CASE = YES	0.94 (0.91, 0.97)	< 0.001
ANESTHESIA DURATION, MINUTES (CHANGE IN HAZARD FROM DURATION 82 TO 195)	0.92 (0.90, 0.94)	≤ 0.001
SEX = FEMALE (VS. MALE)	0.99 (0.95, 1.02)	0.69
AGE, YEARS (CHANGE IN HAZARD FROM AGE 42 TO 68)	0.97 (0.95, 0.99)	0.05
RACE (REFERENCE = ‘WHITE’)		0.53
AMERICAN INDIAN OR ALASKA NATIVE	1.05 (0.77, 1.44)	
ASIAN	1.02 (0.89, 1.16)	
BLACK OR AFRICAN AMERICAN	0.97 (0.93, 1.01)	
UNKNOWN OR OTHER	0.96 (0.89, 1.04)	
HEIGHT, CM (CHANGE IN HAZARD FROM HEIGHT OF 162.6 TO 180.3)	1.01 (0.98, 1.04)	0.32
WEIGHT, KG (CHANGE IN HAZARD FROM WEIGHT OF 69.9 TO 99.8)	1.01 (0.98, 1.03)	0.78
ASA CLASS (REFERENCE = ‘1/2’)		0.01
3	1.00 (0.97, 1.03)	
4/5	0.92 (0.86, 0.97)	
CASE LEVEL (REFERENCE = ‘ELECTIVE’)		< 0.001
LEVEL 1 – MOST URGENT NEED	1.11 (0.93, 1.33)	
LEVEL 2 – URGENT MEDICAL NEED	1.25 (1.14, 1.37)	
LEVEL 3 – LEAST URGENT NEED	1.02 (0.89, 1.18)	
LEVEL 4 – NEXT AVAILABLE REGULARLY SCHEDULED TIME	0.82 (0.51, 1.33)

### Post-Hoc Sensitivity Analysis

Order placement prior to PACU admission was associated with 3% reduced PACU duration (p =  < 0.001), 8% decreased odds of multiple administrations of antiemetics (p = 0.02), and 28% reduced odds of having a higher pain score (p < 0.001) (Table 6).

**Table 6:** Secondary analyses

**SECONDARY OUTCOME**	**PACU MOBILE REMINDER EFFECT ESTIMATE (95% CI)**	**P-VALUE**
TOTAL PACU DURATION, LOG-LINEAR COEFFICIENT	1.06 (1.04, 1.07)	≤ 0.001
INCIDENCE OF 2ND+ LINE ANTIEMETICS, ODDS RATIO (UNORDERED)	1.03 (0.96, 1.11)	0.40
PEAK PACU PAIN SCORE, ODDS RATIO (ORDERED)	1.03 (1.00, 1.06)	0.03

## Discussion

In a large retrospective sample of patients admitted to the PACU at a single academic medical center, we demonstrated an association between a mobile electronic PACU order reminder system and more timely order entry. Use of the system was associated with a greater percentage of patients arriving to the PACU with orders entered and, in those without preexisting orders, was associated with more rapid order entry during the PACU stay. In a post-hoc sensitivity analysis, order placement prior to PACU admission was associated with shorter PACU duration and decreased odds of both higher PACU pain scores and the incidence of multiple administrations of antiemetics.

Interestingly, while the reminder system was associated with increased odds of timely order placement, and on-time order placement was associated with shorter PACU durations, we found that patients whose attending utilized the system had longer median PACU durations than those who didn’t. However, this discrepancy is likely related to an interaction with one or more confounding variables. For example, we found that, other than on-time order placement, PACU length of stay was also associated with sex, race, height, weight, ASA class, case level, anesthesia duration, and provider-specific variables.

In the logistic regression analysis, the following provider-level factors were additionally associated with the placement of PACU orders prior to PACU admission: attending change during the case, in-room provider change during the case, and in-room provider with ordering privileges present during the case. Interestingly, these factors had variable effects on the outcome. A change of attending or the presence of an in-room ordering provider were both associated with greater odds of order placement, possibly because the oncoming ordering provider used the handoff as a cue to place the orders at that time. Conversely, a change in the in-room provider was associated with decreased odds of order placement. Each of these findings may be due to the distribution of responsibility for order placement amongst a group of providers, each of whom may have assumed orders were placed by another team member.

To account for other factors which may have affected the likelihood of timely PACU order placement, we additionally adjusted for anesthesia duration, case urgency, and patient ASA physical status. As expected, sicker patients and higher case urgency were associated with a decreased odds of order placement prior to PACU admission, while longer anesthesia time was associated with increased odds.

Over recent decades, the evidence supporting CPOE and electronic alerts and reminders in varying healthcare environments has grown [5, 6]. In the operating rooms, alerts embedded in anesthesia information management systems (AIMS) have been shown to improve compliance with antibiotic administration, treatment of hyperglycemia [5], and more. The transition to PACU, however, offers an additional opportunity where electronic alerts may provide operational or patient benefit. The added challenge of alerting a provider who is not actively working within the AIMS was overcome, in this case, via mobile device push notifications. Our study demonstrates a proof-of-concept that such technology can successfully reach clinicians and impact their behavior, with the potential to translate this method to other areas of healthcare.

It is also important to note that frequent push notifications can contribute to provider fatigue, leading to increased likelihood of being ignored. We attempted to mitigate these issues by allowing clinicians to add personalized user preferences and opt out of specific types of reminders. While data on changes to user preferences over time are not available, anecdotally our attending anesthesiologists have found this functionality useful.

This study has significant strengths including the inclusion of a large, diverse surgical population at a tertiary medical center operating room. The large sample size allowed increased power to detect significant differences between cases in which an order reminder system was or was not used for PACU order placement. Furthermore, the robust data collection procedures and analytic plan are easily replicated, leading to increased trustworthiness of results.

However, there were also several limitations. First, due to its single-center, retrospective design, the results of this study may not be generalizable to other patient populations. Also, we defined reminder system use as positive if the attending anesthesiologist received at least one push notification on the day of the surgery. We were unable to directly link reminders to individual surgical cases, and so our classification of cases likely included some false positives (cases in which the attending received an alert on the same day as, but not directly linked to, a specific case). Nor were we able to confirm acknowledgement of the reminder by the clinician. As a suggestion for future quality improvement initiatives, a more precise definition of system usage and reminder receipt could eliminate such bias. Similarly, the removal of cases with missing data is another potential source of bias. However, we feel that due to the relatively small number of cases with missing covariate data (< 1%), any bias that may be introduced is negligible.

In conclusion, in this single-center retrospective study, we found that an electronic reminder system was associated with timelier PACU order placement, which in turn may lead to improved patient outcomes.

## Data Availability

No datasets were generated or analysed during the current study.
